# Pregnancy and olfaction: a review

**DOI:** 10.3389/fpsyg.2014.00067

**Published:** 2014-02-06

**Authors:** E. Leslie Cameron

**Affiliations:** Department of Psychological Science, Carthage CollegeKenosha, WI, USA

**Keywords:** self report, odor identification, threshold, hedonics, pregnancy sickness, hormones, hyperosmia, hyperreactivity

## Abstract

Many women report a heightened sense of smell during pregnancy. Accounts of these anecdotes have existed for over 100 years, but scientific evidence has been sparse and inconclusive. In this review, I examine the literature on olfactory perception during pregnancy including measures of self-report, olfactory thresholds, odor identification, intensity and hedonic ratings, and disgust. Support for a general decrease in olfactory thresholds (increase in sensitivity) is generally lacking. There is limited evidence that some suprathreshold measures of olfactory perception, such as hedonic ratings of odors, are affected by pregnancy, but these effects are idiosyncratic. In this review, I explore the hypotheses that have been put forth to explain changes in olfactory perception during pregnancy and provide suggestions for further research.

## INTRODUCTION

Anecdotal reports of heightened sense of smell during pregnancy are common, and the majority of pregnant women report increased olfactory sensitivity (Nordin et al., 2004; Cameron, 2007, 2014). However, the scientific literature on this topic is rather limited and inconclusive. Heightened sense of smell in pregnancy is an important topic because it has been hypothesized to be a trigger for nausea and vomiting (Erick, 1995; Heinrichs, 2002) and an evolutionary mechanism has been proposed – namely that increased olfactory sensitivity protects the developing embryo by reducing the likelihood that the mother will ingest toxins (Steiner, 1922; Profet, 1992). In this review, I summarize the literature on pregnancy and olfaction in humans and explore the possible mechanisms that could underlie the changes women often notice in their perception of odors during pregnancy.

## SELF-REPORTED CHANGE IN SENSE OF SMELL DURING PREGNANCY

The most consistent source of evidence that the sense of smell of women changes during pregnancy comes from anecdotal reports and questionnaire studies. It is clear from perusing websites, reading popular books on pregnancy, and from discussions with pregnant women, that *something* in the perception of odors changes during pregnancy^1^. As early as 1895, Zwaardemaker documented that self-reported hyperosmia is common in pregnancy, although he also noted that empirical measurements of this phenomenon were lacking (Zwaardemaker, 1895). Steiner (1922) reported that almost all pregnant women report a stronger sense of smell, usually in the early months of pregnancy and particularly in the first pregnancy. Henssge (1930) described a case study in which a 27-year-old pregnant woman reported that her olfactory “sensitivity increased” and that odors that were “normally imperceptible were now unbearable.” Henssge (1930) indicated, in that report, that he encountered frequent cases of such “hypersensitivity” in the early phases of pregnancy and although no psychophysical measurements were made, he stated that “Beyond doubt, the patients experienced these odors in response to genuine stimuli which were imperceptible to normal people” ^2^.

According to two more recent studies, approximately two-thirds of pregnant women rate their sense of smell as higher than normal (Cameron, 2007) or as abnormally sensitive (Nordin et al., 2004). A third study also found pregnant women to rate their sense of smell as more sensitive compared to controls, particularly later in pregnancy and even in the postpartum period (Ochsenbein-Kölble et al., 2007). Cameron (2007) found that 85% of pregnant women (*n* = 60) identified at least one odor to which they were more sensitive and Nordin et al. (2004) reported that, relative to non-pregnant women (*n* = 76), more of the pregnant women (*n* = 144) reported “stronger-than-normal smell sensation” of particular odors, including cooking odors, cigarette smoke, spoiled food, perfumes, spices, and coffee. This was particularly evident early in pregnancy. In a subsequent study using the Chemical Sensitivity Scale for Sensory Hypersensitivity (Nordin et al., 2003), Nordin et al. (2007) found self-reported hyperosmia (defined as “increased odor sensitivity during the past month compared to what is normal to that individual” p. 340) in pregnant women (*n* = 95) to be specific to a set of odors, such as cigarettes, prepared or spoiled food, coffee, gasoline, and perfumes.

While the preponderance of self-reports appear to reflect olfactory hypersensitivity, it should be noted that not all studies have found increased self-reported olfactory hypersensitivity in pregnancy. In fact, one early case study described a 25-year-old pregnant woman with asthma who complained of experiencing nearly complete loss of sense of smell (and taste) in early pregnancy, which resolved later in pregnancy (Schmidt, 1925). Moreover, Gilbert and Wysocki (1991) noted in a sample of 13,610 pregnant and 277,228 non-pregnant women who were part of the National Geographic Smell Study, that pregnant women rated their own sense of smell significantly *lower* than non-pregnant women on a 5-point Likert scale. Kölble et al. (2001) reported no significant difference in self-rated sense of smell between 53 pregnant and 59 non-pregnant women. The reason for the disparate data on self-report is unclear, although it does perhaps reflect the idiosyncratic nature of olfaction in general and olfaction during pregnancy in specific.

## HYPEROSMIA

Given that olfaction is important for detecting danger and enjoying food as well as for overall quality of life (Deems et al., 1991; Miwa et al., 2001; Hummel and Nordin, 2005), much research has focused on the causes and impact of loss of sense of smell, either hyposmia or anosmia. Relatively less research has explored heightened sense of smell or hyperosmia. But hyperosmia is important because, even if relatively rare, it is thought to be disruptive to normal functioning (e.g., Erick, 1995; Heinrichs, 2002; Nordin et al., 2005).

Hyperosmia refers to the condition in which there is an increase in olfactory sensitivity. Sensitivity is the inverse of threshold, which in the case of olfaction refers to the minimum concentration of an odor required for its detection. Therefore, an *increase* in olfactory sensitivity is equivalent to a *decrease* in the threshold for detection of an odor. Hyperosmia is relatively infrequently reported and true cases may be relatively rare. There are reports based on empirical testing that hyperosmia occurs in patients with temporal lobe epilepsy (Campanella et al., 1978; Grant, 2005), Addison’s disease (Henkin and Bartter, 1966), and migraines (Hirsch, 1992). However, these findings are controversial. For example, West and Doty (1995) pointed out that there is considerable inconsistency in the epilepsy literature, Murphy et al. (2003) indicated that replications of the findings for Addison’s disease have not been forthcoming and Demarquay et al. (2006) did not find hypersensitivity in patients with migraines. Moreover, patients with specific complaints of “chemical hypersensitivity” have normal olfactory thresholds for those stimuli that have been assessed, namely phenyl ethyl alcohol (PEA, a rose odor) and methyl ethyl ketone (a common solvent; Doty et al., 1988).

It is imperative to stress that most reports of “hyperosmia” or “olfactory hypersensitivity” are anecdotal and lack empirical verification. In light of evidence that self-reported chemosensory function can be unreliable (Nordin et al., 1995; Landis et al., 2003; Soter et al., 2008; Shu et al., 2009) it is important that olfactory sensitivity be measured in cases of suspected hyperosmia. Moreover, what is meant by “heightened sense of smell” or “heightened sensitivity” in the general public may not correspond to the same phenomenon as the hyperosmia defined by olfactory scientists. Steiner (1922) wondered whether the self-reported hypersensitivity might actually be a “subjective” experience.

## HYPEROSMIA IN PREGNANCY?

Given that the self-report data suggest the presence of hyperosmia in pregnancy, it is important to distinguish between the measures used to assess olfaction in pregnant women, some of which, at least on the surface, do not appear to measure sensitivity *per se*. In general, it has been assumed that “heightened olfactory sensitivity” or “hyperosmia” refers to reduced olfactory detection thresholds, although this, in fact, need not be the case. This section reviews the literature on olfactory detection and recognition thresholds.

### DETECTION THRESHOLDS

Several studies have examined the effect of pregnancy on olfactory detection thresholds. Kölble et al. (2001) found no significant difference in olfactory detection thresholds between non-pregnant women and women in the first trimester of pregnancy^3^. Thresholds were measured with the odor *n*-butanol, which has a window-cleaner like smell, using a staircase procedure in which the target odor had to be selected from triplets of stimuli (two “blanks” and one odorant). Savovic et al. (2002) measured olfactory detection thresholds for six odors, namely anethol (aniseed), vanillin, PEA, citral, menthol, and pyridine (a fishy odor), in 20 non-pregnant and 20 women in their first trimester of pregnancy using the Fortunato–Niccolini air-dilution olfactometer (Caruso et al., 2001). Thresholds were determined by the smallest volume of air, presented during normal inspiration, that resulted in the detection of an odor. There were no significant differences between the detection thresholds of pregnant and non-pregnant women. Laska et al. (1996) measured olfactory detection thresholds longitudinally across all three trimesters and found no significant systematic changes across trimesters, nor between the 20 pregnant and 20 non-pregnant women, although compared to controls, pregnant women’s thresholds were significantly higher in the first trimester and significantly lower in the third trimester. Laska et al. (1996) also used the odorant *n*-butanol, but with a single ascending staircase technique. The finding from Laska et al. (1996) is consistent with Good et al. (1976) who, in a case study, found that the number of *false alarms* (responding that the musk-like compound Exaltolide was present when it was not) decreased as the woman came closer to parturition. Therefore, her *d*′ (a measure of sensitivity derived from signal detection theory; see Green and Swets, 1966) was higher in the third than the second trimester. Ochsenbein-Kölble et al. (2007) also showed that olfactory detection thresholds for *n*-butanol decreased over the course of pregnancy in 39 women and were statistically lower in the last trimester and postpartum than that of 45 non-pregnant controls. While the decrease in detection threshold in late pregnancy is consistent with Laska et al. (1996) and Good et al. (1976), the postpartum results are surprising and are not consistent with other reports in the literature on olfactory thresholds in the postpartum period (see Recognition Thresholds). More recently, Cameron (2014) measured detection thresholds for PEA longitudinally across the three trimesters of pregnancy in 23 women and found no significant differences in detection threshold between pregnant women and 25 non-pregnant controls. This study employed the standard 1-up, 2-down staircase method, as described by Doty (2000).

**FIGURE 1:**
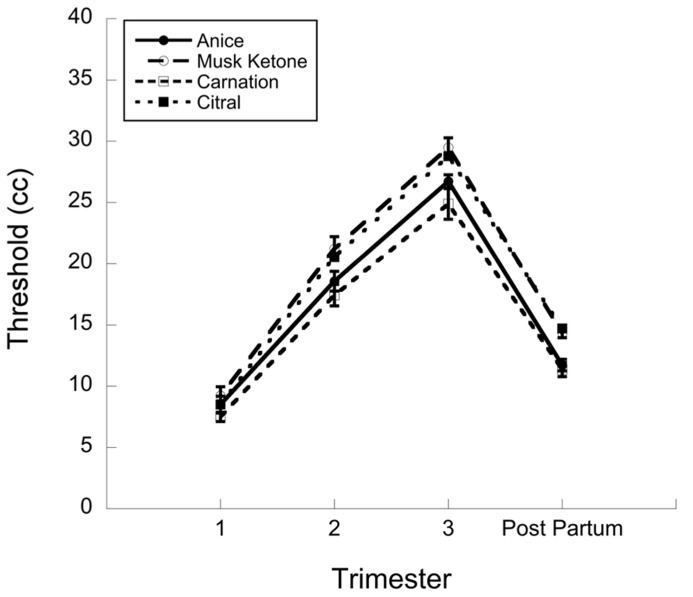
**Data from Luvara and Murizi (1961).** Threshold (in cubic centimeter, as measured by the blast injection technique) across pregnancy trimester and postpartum. There were a total of 47 participants; 14–21 per session and some participated in more than one session.

The only study in the literature that clearly demonstrated a significant decrease in olfactory detection thresholds in early pregnancy was conducted by Luvara and Murizi (1961). For each of four odors (anise, musk ketone, carnation, and citral), the authors established detection thresholds using the blast-injection technique (Elsberg and Levy, 1935). There were 47 women tested in this study, some of whom were tested twice (in two phases of pregnancy or during pregnancy and postpartum). I have plotted the data, provided only in tabular format in the original article, in **Figure 1**. Doty (1976) previously conducted statistical analyses of these data and reported that all comparisons were significant.

Of particular interest, with respect to the purported heightened sensitivity in early pregnancy, is that there is a significant difference in thresholds between the first trimester and the postpartum period. To my knowledge, this constitutes the only empirical support in the literature for lower olfactory detection thresholds in early pregnancy^4^. However, the blast-injection technique, unlike other measures of threshold, may reflect changes in nasal engorgement in the later stages of pregnancy (see Pregnancy and the Nose).

It is worthy of note that all of the studies that have measured olfactory detection thresholds in pregnant women have employed validated methods for measuring thresholds; these methods are sensitive to differences in smell function between sexes and age groups (Doty et al., 1984a) and can identify some clinical populations, such as patients with Alzheimer’s and Parkinson’s (Doty, 2003). Thus, failure to observe changes in olfactory detection thresholds in pregnant women is unlikely due to the method employed. However, some cases of increased sensitivity to odors have been demonstrated using sensitive *signal detection* measures. For example, Doty et al. (1981) used such methods to demonstrate subtle changes in olfactory sensitivity across the menstrual cycle. Cameron (2014) adopted the same method as Doty et al. (1981) to measure olfactory sensitivity in pregnant women. After the assessment of their olfactory detection threshold, participants completed an additional 75 signal detection trials, using an odorant whose concentration was close to the participant’s own threshold. On each trial two jars were presented – on half of the trials one of the jars contained the weak PEA odorant (“signal + noise”) and the other the diluent alone (“noise”) and on the other half of the trials both jars contained the diluent alone (“noise”). In this method, *hits* refer to trials in which the *signal* was present and the participant said it was and *false alarms* refer to trials in which the *signal* was not present but the participant said it was. Hits and false alarms were used to compute *d*′ (sensitivity) and c (response bias)^5^. Cameron (2014) employed this signal detection paradigm, albeit with a smaller number of trials than used by Doty et al. (1981), and still found no significant increase in olfactory sensitivity (i.e., no increase in *d*′) in pregnant women. The data suggest that pregnant women exhibit a more *liberal* criterion (i.e., made more *false alarms*) early in pregnancy, although the difference between pregnant and non-pregnant women was not statistically significant in this small sample. A more liberal criterion would be consistent with the greater number of *false alarms* reported in Good et al.’s (1976) case study.

In summary, there is only limited evidence for decreased in olfactory detection thresholds (hyperosmia) in pregnant women, even using sensitive measures and despite the self-reported increase in sensitivity.

### RECOGNITION THRESHOLDS

**FIGURE 2:**
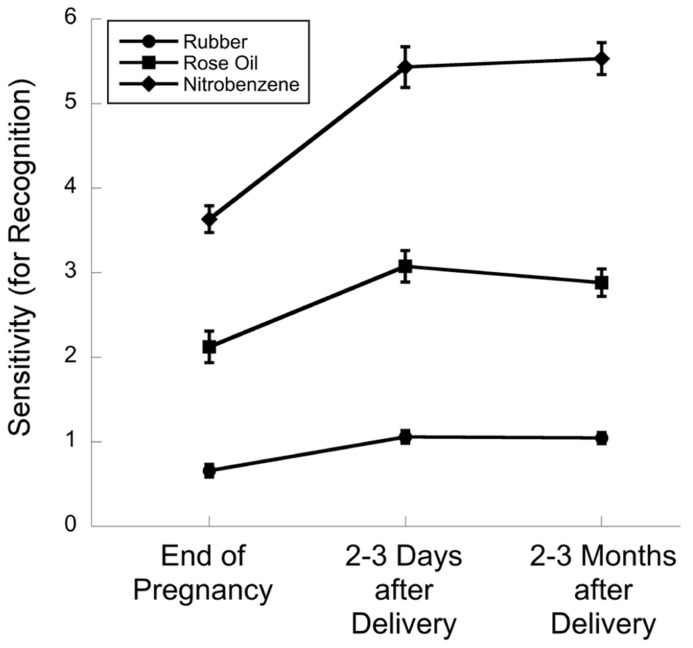
**Data from Hansen and Glass (1936).** Sensitivity (inverse of threshold) for recognition for each of three odors, plotted for the end of pregnancy and two postpartum test sessions. Zwaardemaker olfactometer was used to measure thresholds of 22 participants who were followed longitudinally.

Two studies have measured olfactory recognition thresholds in pregnant women. Hansen and Glass (1936), using a Zwaardemaker olfactometer and a method of ascending limits, tested 22 women and found that recognition sensitivity was lower at the end of pregnancy compared to two postpartum periods (2–3 days or 2–3 months after delivery) for all three odors tested (rubber, rose oil, and nitrobenzene (bitter almonds)). I have plotted these data in **Figure 2**. Doty (1976) reported that the differences between the thresholds in the two postpartum periods were not statistically significant, but that they were both significantly lower than thresholds at the end of pregnancy.

**FIGURE 3:**
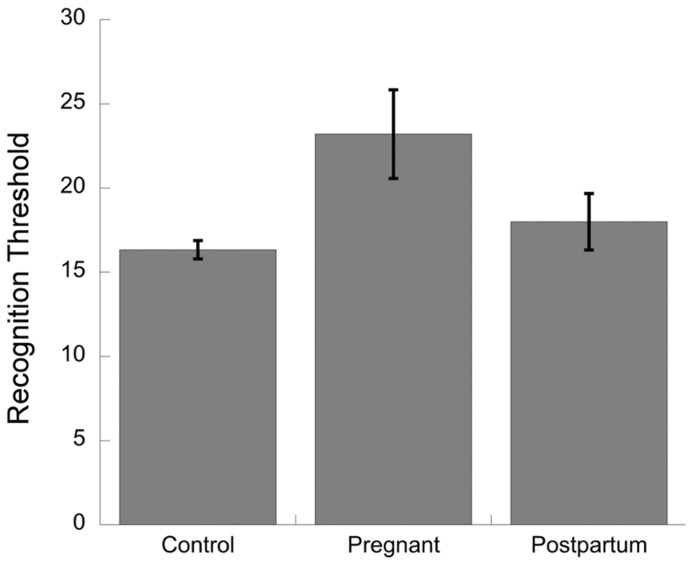
**Data from Noferi and Giudizi (1946).** Recognition thresholds for non-pregnant, pregnant, and postpartum women. Blast olfactometer was used to determine recognition thresholds for lemon. There were 15 participants per group in a cross-sectional design.

Noferi and Giudizi (1946) compared recognition thresholds for a lemon odor using the blast-injection technique in a cross-sectional study. **Figure 3** shows that thresholds were significantly higher in 15 women in late pregnancy compared to 15 non-pregnant controls and compared to 15 women who were within 2 weeks postpartum (Doty, 1976). Again, this may be due to the method of testing.

In summary, the data on recognition thresholds suggests that late pregnancy is a period of low sensitivity (recognition thresholds are *high*) relative to the postpartum period. These results are inconsistent with the detection threshold results from Cameron (2014), Good et al. (1976), and Laska et al. (1996) but are consistent with a more recent report of decreased threshold sensitivity in the third trimester compared with controls (Ochsenbein-Kölble et al., 2007, using the same methods as Kölble et al., 2001).

## OTHER MEASURES OF SMELL FUNCTION IN PREGNANCY

The inconsistency between the self-reported increased olfactory sensitivity in pregnant women and the lack of evidence of decreased olfactory (detection or recognition) thresholds begs the following questions: How is olfactory processing affected by pregnancy? Do pregnant women outperform non-pregnant women on other olfactory tasks, such as odor identification? And do pregnant women rate the intensity and hedonicity of odors differently than non-pregnant women? This section reviews the literature on the effect of pregnancy on several measures of olfaction other than thresholds.

### ODOR IDENTIFICATION

Eight studies have assessed odor identification in pregnant women (Gilbert and Wysocki, 1991; Laska et al., 1996; Kölble et al., 2001; Savovic et al., 2002; Swallow et al., 2005a; Cameron, 2007; Ochsenbein-Kölble et al., 2007; Kim et al., 2011).

Gilbert and Wysocki (1991) compared odor identification in pregnant and non-pregnant women using six odors – isoamyl acetate (banana/pear), eugenol (the primary component of clove oil), rose, a mixture of mercaptans (smell added to natural gas), galaxolide (musky), and androstenone (musky/urine). Participants were instructed to scratch and sniff the odor and then to select one of the following words that best described the odor: no odor, floral, musky, urine, foul, ink, spicy, woody, fruity, burnt, sweet, and other. They found no significant general effect of pregnancy status on odor identification, except that pregnant women were able to identify clove significantly more readily. Laska et al. (1996) examined odor identification for 12 odors: all of the odors employed by Gilbert and Wysocki (1991) except for the mixture of mercaptans, as well as citronelle nitrile (lemon), peanut aroma, Chanel No. 5, anethole, linalool (lavender), n-butanol (described by the authors as oily, alcoholic) and a 12-component mixture. Participants sniffed the odors presented in squeeze bottles and were instructed to generate a name or attempt to describe the odor^6^. Despite different methods, the results were consistent with Gilbert and Wysocki (1991) in that pregnant women outperformed non-pregnant women in identifying eugenol. However, they were *less* able to provide appropriate descriptors or accurate names for peanut, banana, aniseed, and lemon.

Kölble et al. (2001) and Ochsenbein-Kölble et al. (2007) measured odor identification using the 16-item Sniffin’ Sticks (odors include orange, peppermint, turpentine, cloves, leather, banana, garlic, rose, fish, lemon, coffee, anise, cinnamon, liquorice, apple, and pineapple). Kölble et al. (2001) found that, relative to controls, women in the first trimester of pregnancy tended to perform more poorly and Ochsenbein-Kölble et al. (2007) found no significant change across pregnancy status compared to controls. No data were presented as to the relative ability to identify specific odors. Consistent with these studies, Kim et al. (2011) reported no significant difference between 35 pregnant and 40 non-pregnant women using the Korean Version of the Sniffin’ Sticks (KVSS-II test) and Savovic et al. (2002) found no significant difference in odor identification performance of women in their first trimester compared to controls using the Fortunato–Niccolini olfactometer. Swallow et al. (2005a) tested odor identification for six odors (three “safe” – strawberry, vanilla, and melon and three “potentially harmful” – coffee, cabbage, and fish) and found no significant difference in odor identification among three groups – pregnant women (*n* = 55), non-pregnant women (*n* = 42), and men (*n* = 48) – except for the strawberry odor. Non-pregnant women outperformed pregnant women and men, but correct identification overall for strawberry was relatively poor and worse than for other odors (Swallow, personal communication). Finally, Cameron (2007) measured odor identification in pregnant women (20 in each trimester), 20 non-pregnant controls and 20 women in the postpartum period on the 40-item scratch and sniff University of Pennsylvania Smell Identification Test (UPSIT; Doty et al., 1984b) and found no overall effect of pregnancy status on odor identification. However, watermelon was identified significantly better by pregnant women^7^.

In summary, odor identification has been explored in pregnant women using a wide range of odors, with several methods, and in a number of different cultural contexts. There is no evidence that pregnant women generally identify odors consistently better than non-pregnant controls. In fact, some studies have even reported a tendency for *worse* performance in pregnancy, at least for some odors (Laska et al., 1996; Kölble et al., 2001; Swallow et al., 2005a). Notwithstanding these negative findings, there is evidence that pregnant women identify some odors better than controls [clove by Gilbert and Wysocki (1991) and Laska et al. (1996); strawberry by Swallow et al. (2005a), and watermelon by Cameron (2007)], suggesting that perhaps there is an improved ability to identify some odors during pregnancy.

### INTENSITY RATINGS

Olfactory perception in pregnant women has also been assessed by means of odor intensity ratings. Gilbert and Wysocki (1991) found that two odors (isoamyl acetate and a mixture of mercaptans) of six were rated as significantly more intense by pregnant women compared to controls, but they also found that two other odors (androstenone and galaxolide) were rated as significantly less intense by pregnant women compared to controls. Likewise, Cameron (2007) found that overall there was a trend for pregnant women, compared to controls, to rate odors as more intense in the first trimester (~75% of odors were rated as slightly more intense by pregnant women), but there was a statistically significant increase in intensity ratings for only three (leather, lemon, and natural gas) of 39 UPSIT odors.

Laska et al. (1996) reported that intensity judgments were relatively stable across test sessions and consistent between pregnant and non-pregnant women. Pregnant women rated only two (galaxolide and androstenone) of 12 odors to be statistically significantly more intense, but this was not consistent, nor stable across pregnancy. Kölble et al. (2001) and Ochsenbein-Kölble et al. (2007) had pregnant women rate the intensity of 10 common odors (deodorant, bacon, clove, cigarette butt, coffee, androstenone, acetic acid, rum, peanut butter, and chocolate). There were no statistically significant differences in the intensity ratings between pregnant women and controls in either study. Swallow et al. (2005a) found no overall difference between groups in ratings of odor “strength,” although melon was rated to be statistically significantly stronger by pregnant women compared to non-pregnant women and men.

In a questionnaire study, Nordin et al. (2004) found the percentages of “stronger-than-normal sensations” to be high for women in the first two trimesters of pregnancy for most of the 14 odors investigated. It must be noted, however, that this was a self-report measure, and not one based on rating of odors that were being smelled at the time of testing.

In summary, although overall odor intensity ratings do not appear to be higher in pregnant than non-pregnant women, there is some evidence that odor intensity ratings for select odors are higher in pregnant women than in controls.

### HEDONICS

Another metric of olfactory perception that has been employed to assess the impact of pregnancy on olfaction is hedonic or pleasantness ratings of odors. Six studies have examined the rating of odor hedonics in pregnancy (Gilbert and Wysocki, 1991; Laska et al., 1996; Kölble et al., 2001; Nordin et al., 2005; Swallow et al., 2005a; Cameron, 2007; Ochsenbein-Kölble et al., 2007). Gilbert and Wysocki (1991) reported that half of the odors they tested (galaxolide, eugenol, and mercaptans) were rated as significantly less pleasant by pregnant women and Kölble et al. (2001) reported that pregnant women found cigarettes, coffee, and rum to be significantly less pleasant than controls, although there were no differences between the groups for hedonic ratings of other odors. Ochsenbein-Kölble et al. (2007) reported that, compared to controls, pregnant women rated cloves and coffee to be less pleasant during pregnancy although the differences in ratings for coffee were only statistically significant in the first trimester. Cameron (2007) reported there was a tendency for pregnant women to rate most odors on the UPSIT as less pleasant than controls. Orange, grape, and natural gas were rated as significantly less pleasant by pregnant women compared to controls. Swallow et al. (2005a) reported that overall pregnant women rated odors to be significantly less pleasant than did men but that there were no specific odors that accounted for the result. Laska et al. (1996) reported considerable variability in hedonic ratings in pregnant women. Only peanut was statistically significantly rated to be less pleasant by pregnant women across all trimesters of pregnancy. There was no consistent pattern across the remainder of the odors.

There are relatively few studies that report that pregnant women rate odors as more pleasant. Compared to the odors that are rated as less pleasant, there are relatively fewer odors that are rated as more pleasant, and the results are not consistent across pregnancy. Gilbert and Wysocki (1991) reported that androstenone was rated as significantly more pleasant in pregnant women (pregnancy phase not known). Cameron (2007) reported that only one of 39 odors (fruit punch) was rated to be marginally more pleasant in the first trimester of pregnancy, and Laska et al. (1996) indicated that clove, aniseed, and perfume were rated as significantly more pleasant in some trimesters (this varied with odor). Ochsenbein-Kölble et al. (2007) found that acetic acid was rated as significantly more pleasant during the second and third trimesters of pregnancy.

In addition to rating pleasantness, some studies have asked pregnant women to identify odors that they find particularly pleasant or unpleasant. Cameron (2007) reported that 90% of pregnant women identified odors that they found to be less pleasant. In addition to a range of food odors (e.g., meat, fish, and eggs), pregnant women indicated that noxious odors such as cigarettes, fumes, and garbage were particularly unpleasant. They also reported that some “social odors,” such as body odor, baby odors, and perfume and colognes were unpleasant^8^. Cameron (2007) also reported that less than half as many odors were identified by pregnant women as being *more* pleasant, the vast majority of them being foods (e.g., pickles, fruits, and spices). It is worthy of note that Steiner (1922) quoted several women who cited many of these same items – e.g., burnt, spoiled or cooked food, cigarette smoke, and perfume – as being unpleasant, particularly during the early stages of pregnancy.

It is clear from the above that most studies have demonstrated changes in odor hedonics during pregnancy, typically resulting in a reduction in the ratings of pleasantness of odors, although this depends on odor. Anecdotally, pregnant women indicate that the hedonics of odors change, specifically that odors smell bad or that they are particularly aware of foul odors (see text footnote 1).

## DISGUST

People’s beliefs about the potential danger of exposure to certain chemicals and odors may be a factor that contributes to disgust. Rozin and Fallon (1987) defined disgust as “revulsion at the prospect of oral incorporation of offensive objects. These objects have contamination properties” (p. 23). To the extent that odors are related to these “offensive objects,” they could be considered to be a source of contamination.

The finding that many of the odors that are identified as less pleasant during pregnancy are food related odors or “noxious” substances, such as cigarettes and smoke, is consistent with the idea that these odors could be thought by pregnant women to be contaminants. Moreover, given that there is a change in odor hedonics in pregnancy, it seems likely that pregnant women would score particularly high on a measure of disgust. Fessler et al. (2005) administered the Disgust Scale (Haight et al., 1994) to 496 pregnant women and reported that women in the first trimester scored significantly higher on this scale compared to the last two trimesters of pregnancy.

## CLINICAL OR EVOLUTIONARY RELEVANCE

The consistent finding that pregnancy affects the hedonic valence of odors and the finding that disgust sensitivity is high, particularly early in pregnancy, leads to two important clinical and evolutionary questions: What is the relationship between olfaction and nausea and vomiting? And is there support for the embryo protective hypothesis?

### HYPEROSMIA AND NAUSEA AND VOMITING IN PREGNANCY

Nausea and vomiting (“morning sickness”) afflicts about three-quarters of pregnant women (e.g., Lacroix et al., 2000; Niebyl, 2010). The idea of a causal link between increased olfactory sensitivity and nausea and vomiting is compelling (e.g., Erick, 1995; Heinrichs, 2002; Niebyl, 2010). Such a link could be important for understanding and managing maternal nutritional status, which has a significant impact on fetal well-being and development. However, this link depends on a heightened sense of smell, which has yet to be documented. Nonetheless, Heinrichs (2002) reported a substantial decrease in reports of incidence of nausea and vomiting in pregnant women with congenital anosmia (only one of nine patients). Moreover, Cantoni et al. (1999) reported that 58% of 500 women responded that there were odors that caused nausea during pregnancy and Swallow et al. (2005b) found that, in a sample of 273 pregnant women, those who were adversely affected by odors scored higher on a measure of the severity of their nausea and vomiting. However, Hummel et al. (2002) found no significant correlation between the incidence of self-reported nausea and vomiting and performance on olfactory detection threshold, discrimination nor identification tasks in 53 women in the first trimester of pregnancy. The authors suggested that nausea and vomiting may not be strongly tied to basic olfactory function.

Classical conditioning could explain the relationship between the perception of odors and nausea and vomiting in pregnancy. Perhaps pregnant women rapidly condition to odors that are present during a moment of nausea and/or vomiting, as in the Garcia effect (conditioned taste aversion). Thus, a previously neutral, conditioned stimulus (an odor) becomes associated with an unconditioned stimulus (whatever instigated the nausea/vomiting) and the conditioned response of nausea/vomiting becomes elicited by the conditioned stimulus (the odor). Subsequent exposures to that neutral odor could invoke a rapidly conditioned response (nausea and vomiting). An important aspect of this hypothesis is that it does not require hyperosmia. The odor could be present and perceived at essentially any intensity level. Note that in a study published only in abstract form, Bartoshuk and Wolfe (1990) reported conditioned aversion that was induced by smell, but not by taste.

### THE EMBRYO PROTECTIVE HYPOTHESIS

It has been argued that hypersensitivity to odors would provide a protective function for the embryo by limiting what the mother ingests, particularly early in pregnancy when the embryo/fetus is most vulnerable. This notion was proposed as early as 1922 by Gabriel Steiner (Steiner, 1922) and elaborated more recently by Margie Profet (Profet, 1992). The hypothesis is that hyperosmia in pregnancy leads to nausea and vomiting and that this provides a protective function for the embryo, inhibiting the pregnant woman from ingesting teratogens during the phase of pregnancy when the embryo is most vulnerable (the first trimester).

This hypothesis has two significant limitations. First, the evidence for hyperosmia in pregnancy is weak, as demonstrated in this review. Thus, whatever changes occur in the olfactory system during pregnancy, it is does not appear to result in a generalized lowered detection threshold. Therefore, it seems unlikely that hyperosmia underlies the nausea and vomiting that would protect the embryo. Second, two studies have directly tested this hypothesis and neither one support it. Swallow et al. (2005a) explored odor ratings of liking, strength, and pleasantness for six odors, half of which were considered to be potentially dangerous. Pregnant women did rate odors as significantly less pleasant than non-pregnant women or men. However, there was no significant interaction between group and type of odor (safe or potentially harmful), which would have indicated that pregnant women were more averse to potentially harmful odors. Likewise, Brown et al. (1997) explored the relationship between the intake of bitter vegetables and other foods thought to be harmful (Profet, 1992) and the incidence of nausea and vomiting in a very large sample (*n* = 549). There were no significant differences in the intake of food thought to be harmful to the developing embryo between the group who had nausea and/or vomiting in early pregnancy and the group that did not.

## MECHANISMS UNDERLYING CHANGES IN OLFACTION DURING PREGNANCY

Although the data do not support a general hyperosmia, there does appear to be a change in the perception of odors during pregnancy. Several mechanisms have been suggested to account for this result.

### HORMONES AND SENSE OF SMELL

**FIGURE 4:**
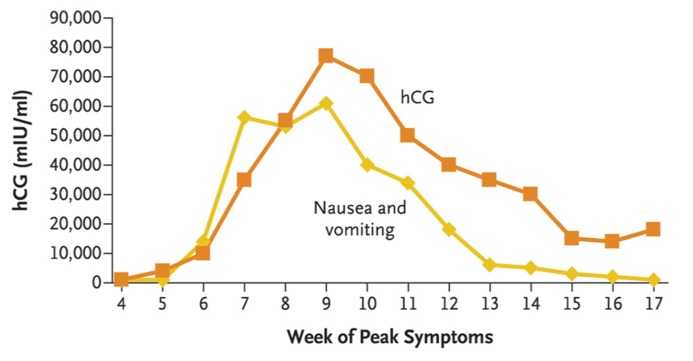
**Human chorionic gonadotropin (hCG) levels (and reports of nausea and vomiting) as a function of number of weeks of pregnancy.** hCG level peaks during the first trimester. From Niebyl (2010), permission received.

Levels of circulating gonadal hormones are often proposed as an explanation for heightened sense of smell. For example, hormone levels are widely believed to explain sex differences, changes in olfactory sensitivity across the menstrual cycle and for the purported changes in olfactory processing in pregnancy (for a review, see Doty and Cameron, 2009). Although olfactory detection thresholds are correlated with circulating levels of estrogen in normally cycling women, thresholds also vary similarly across the menstrual cycle in women taking oral contraceptives, calling into question whether this relationship is causal (Doty et al., 1981). Estrogen levels rise throughout pregnancy, reaching their peak shortly before parturition (Gard, 1998). Thus, one would predict that smell function should improve across pregnancy if estrogen, alone, were involved. This is neither what is observed in measures of olfactory perception, nor what is expected based on self-report. To the extent that one can rely on self-report, which indicates the largest changes in odor perception (particularly odor hedonicity) occur early in pregnancy, the changing levels of the hormone human chorionic gonadotropin (hCG) match the temporal profile of the self-reported changes (Gard, 1998; Niebyl, 2010; and see **Figure 4**). Thus, hCG might be considered to be a candidate underlying changes in olfactory perception, or at least changes in odor hedonicity. Interestingly, incidents of nausea and vomiting are also correlated with hCG levels in pregnancy (see **Figure 4**).

A potentially related condition to the experience of pregnant women’s sense of smell and its relationship to hCG comes from people who are on the controversial hCG hormone diet. Developed by Simeons in the 1950s and sometimes recommended for treatment of obesity, this extremely low calorie diet (500 calories/day) is coupled with intramuscular injections of hCG. The hormone is thought to suppress hunger and allow people to remain on the diet for over a month. This diet gained popularity in the 1970s and had a resurgence several years ago. In the United States, the Food and Drug Administration has warned against the use of this diet because there are no scientific studies that have verified its effectiveness^9^ and the Obesity Society recently published a position statement indicating that they do not condone its use^10^.

People who are injected with hCG as part of this controversial diet and women who are injected with hCG for infertility treatment report, anecdotally, that their sense of smell is heightened. A perusal of blog postings indicates that the sort of self-report of this experience is very similar to the reports of some pregnant women, particularly in early pregnancy. For example, several people posted on HCG DIET INFO FORUMS (August 29, 2010):

⋯ about the heightened sense of smell ⋯ but I couldn’t sleep on my left side last night because I could smell my husband’s breath and I couldn’t sleep on my right side because I could smell a sealed bottle of incense I had in my bedside table’s drawer. It’s ridiculous!!

I thought it was just me with the extra sensitive nose lately. I’ve always had a good “sniffer” but lately I smell everything!

I feel like a superhero or something with this new sense of smell and it is making me crazy!

These sorts of comments are reminiscent of comments by pregnant women, including the one reported at the start of this review^1^. Here are two responses to that posting:

I’ve always had a sensitive nose and it was magnified by my pregnancy as well. Horrible. I sometimes find being out in public overwhelming with all the perfumes and body odors and whatnot.

My second pregnancy was a[n] olfactory nightmare. The dog stunk to high heaven, my firstborn was a diaper-wearing terror of wafting fumes, and I actually woke my husband up from a sound sleep to make him go brush his teeth in the middle of the night. Really.

To my knowledge, no study has examined smell function in people on the hCG diet. Moreover, no study has measured hormone levels and smell function concomitantly during pregnancy, but the evidence so far does not suggest a strong correlation between estrogen and hyperosmia.

### COGNITIVE/ATTENTIONAL MECHANISMS

Another possible explanation for the change in odor perception during pregnancy is that the effect is a more cognitive (high-level) than sensory (low-level) one. Such a high-level change in odor processing would not be expected to result in changes measured by most standard tests of smell function. Evidence for a high-level mechanism comes from event-related potential (ERP) data. Olofsson et al. (2005) measured chemosensory ERPs in 15 pregnant and 15 non-pregnant women and found no significant differences between groups in amplitude nor latency of N1 and P1 components (which reflect sensory processing), but rather a tendency for shorter latency and higher amplitude of the more perceptual/cognitive P3 component in the pregnant group. This suggests that changes may be observed for more central levels of olfactory processing. This is consistent with the results reported above that show that relative to later in pregnancy, pregnant women exhibited a more liberal criterion in an odor detection task using a signal detection paradigm in early pregnancy (Cameron, 2014).

It is worthy of note that pyridine, which has a trigeminal component, was used as the stimulus in Olofsson et al.’s (2005) study [and in the previously mentioned Broman et al.’s (2003) study that showed significantly reduced thresholds in pregnancy] and it has been suggested that perceived hyperosmia may be related to trigeminal function (Nordin et al., 2005). In addition, pyridine is an unpleasant odor, which may also have been a factor in the outcome of these studies.

### HYPERREACTIVITY

The cognitive hypothesis is consistent with a hyperreactivity hypothesis: self-reported olfactory hypersensitivity in pregnant women could reflect a hyper-*awareness* of or irritation produced by many odors. This may be analogous to the literature on hyperosmia in migraines, as described by Demarquay et al. (2006) “In the field of migraine and MCS [multiple chemical sensitivity], this term [hypersensitivity or hyperacuity] is used in a broader sense, reflecting the discomfort perceived by the patient as an inappropriate and excessive odour-induced response.” (Demarquay et al., 2006, p. 1128). Steiner (1922) suggested that perhaps the self-reported increased sensitivity in pregnancy was in fact an emotional reactivity. There is some evidence of this from questionnaire studies. Nordin et al. (2005), 2007 found that pregnant women, particularly in the first trimester of pregnancy, score higher on the Chemical Sensitivity Scale for Sensory Hyperreactivity (Nordin et al., 2003). This lead the authors to conclude that “pregnant women to a large degree are affected by odorous/pungent substances in their daily activities” (Nordin et al., 2007, p. 341). They also conclude that olfaction is the major contributor to this sensory hyperreactivity, and that this hyperreactivity does not extend to auditory stimuli.

The general decrease in pleasantness of odors during pregnancy may result in a change in the *awareness* of or *attention* to odors. Bad smells attract our attention. The awareness that is drawn to the odors may be incorrectly interpreted by pregnant women as hyperosmia. This is consistent with the correlation between self-rating of olfactory function and self-rating of odor annoyance in a sample of 1311 people (Knaapila et al., 2008).

Such a hyperreactivity or hyperawareness may be under relatively high-level, cognitive control. Dalton (1996) demonstrated that when participants were exposed to the odor isobornyl acetate (balsam) and told that the odor was a “natural, healthy extract,” they adapted to it and rated its perceived intensity to be low and decreasing across exposure duration. On the other hand, when participants were exposed to the same odor and told that it was “potentially hazardous” they became sensitized to it and rated its perceived intensity to be relatively high, particularly toward the end of the exposure duration. Interestingly, detection thresholds remained constant, regardless of the nature of the information given. Risk perception appears to influence perceived odor intensity. Therefore, one possible explanation of self-reported olfactory hypersensitivity in pregnant women is that it reflects a hyperreactivity to odors that arises from beliefs about health risks associated with odors. Interestingly, beliefs about the health risks of exposure to certain odors may or may not occur at the level of conscious awareness (Dalton, 2012).

### PREGNANCY AND THE NOSE

Although the first trimester appears to be the time during which the greatest changes in perception of odors occur, some of the detection and recognition threshold data reported above suggested impaired olfactory function at the end of pregnancy (Hansen and Glass, 1936; Noferi and Giudizi, 1946; Luvara and Murizi, 1961; Ochsenbein-Kölble et al., 2007). This may be accounted for by peripheral mechanisms. For example, nasal airflow varies as a function of pregnancy status. As with many tissues of the body the nose becomes more engorged and “stuffy” during pregnancy (Bende and Gredmark, 1999; Ellegard and Karlsson, 1999; Philpott et al., 2004). Nasal congestion occurs in the late stages of pregnancy and thus airflow is reduced, which reduces the ability to perceive odors.

## SUMMARY AND SUGGESTED FURTHER RESEARCH

In this review, I have described all of the extant data on the effect of pregnancy on olfaction. There is no evidence for a general hyperosmia during pregnancy, although it must be noted that there remains a dearth of conclusive studies on this topic. This is surprising given the abundant anecdotal evidence. Therefore, it may be premature to draw strong conclusions.

Several aspects of olfaction and pregnancy require further study. Perhaps the central issue for further study is the effect of odorant-specificity on olfactory perception in pregnant women. Performance on a range of olfactory tasks depends upon the specific odors presented. Further research is necessary to explore this phenomenon in more detail, with carefully selected odors. First, detection and recognition thresholds and odor identification should be measured using a broader range of odors, taking into consideration the hedonic tone of the odors. Second, given the substantial individual differences in odor preference, further research is needed to explore whether there are odors that are commonly reported to be unpleasant by pregnant women (some evidence suggests that there are). Third, intensity ratings for a range of odors at a range of concentrations should be established. Finally, it is important to distinguish between odors that are purely olfactory and those that contain a trigeminal component. The differences in the processing of pyridine by pregnant women in the studies by Olofsson et al. (2005) and Broman et al. (2003) suggests that pregnancy may modify the processing of trigeminal stimuli. This idea deserves further investigation.

Pregnant women have been tested on both low-level threshold (detection) tasks and high-level suprathreshold (identification) olfactory tasks, but further research is needed using both types of task. It is important to distinguish between sensory and cognitive changes in the olfactory system that may be brought about by pregnancy. First, odor detection across a range of concentrations using the method of constant stimuli would enable an examination of differences between psychometric functions (e.g., differences in slopes) of pregnant and non-pregnant women. Second, suprathreshold measurements, such as cross-modal matching, could reveal differences that have not been demonstrated with more common methods of measuring olfactory perception. Future studies could examine performance on tasks that require olfactory cognition, such as tests of odor memory or attention.

Further research is needed to examine the complex relationship between hormones and smell function, particularly with respect to pregnancy. No study has measured hormone levels and smell function concomitantly in pregnant women, but the evidence so far does not suggest a clear and causal relationship between estrogen and hyperosmia given the discrepancy between the self-reported smell function during early pregnancy and the relatively lower levels of estrogen at that time in pregnancy. hCG is thought to stimulate the production of estrogen (Niebyl, 2010) and it is possible that there is a complex interaction among hormones that underlies olfactory perception, particularly in pregnant women.

It is compelling to suppose that there is a link between odors and the onset of nausea and vomiting in pregnancy. At present there is no scientific evidence for a direct link, and yet many women can identify odors that bring on nausea and vomiting. It is worthy of note that nausea is correlated with ratings of food disgust (Fessler et al., 2005) and nausea and vomiting is less common in people with anosmia or hyposmia than in normosmics. Clearly more study is needed in this area. A better understanding of the relationship between olfaction and nausea and vomiting in pregnancy could help the many women who suffer from these symptoms.

## Conflict of Interest Statement

The author declares that the research was conducted in the absence of any commercial or financial relationships that could be construed as a potential conflict of interest.

## Footnotes

1 A sample blog post: ⋯*But there have been some changes [in pregnancy]. Namely, smell. I smell everything to an acute degree bordering on insanity. Let me clarify that. I smell everything bad and it’s making me feel like a crazy woman! I smell garbage, gas, poo, chickens, old eggs, stinky breath, dirty sheets. But flowers and nice perfume? Nah, can’t smell that.* (From http://www.rurallyscrewed.com).

2 All quotes from English translation of the abstract.

3 Sample sizes are reported only once for studies that are discussed in multiple sections of this review. For example, sample sizes for this study were provided in Section “Self-reported Change in Sense of Smell During Pregnancy.”

4 It is worth noting that two unpublished works (Dastur, 2001; Broman et al., 2003) found decreased odor thresholds in pregnancy. Dastur (2001) reported, in a doctoral dissertation, that detection thresholds for PEA were significantly lower in 19 women tested longitudinally across all trimesters of pregnancy compared to 18 non-pregnant controls and the pregnant women tested in the postpartum period. Broman et al. (2003), in an abstract reported at the Association for Chemoreception Sciences, found decreased thresholds for pyridine (a fishy odor) in 30 women in the second trimester of pregnancy compared to 30 non-pregnant women.

5 Sensitivity (*d*′) = *Z* (hit rate) - *Z* (false alarm rate); response bias (*c*) = -0.5 [*Z* (hit rate) - *Z* (false alarm rate)].

6 No information is provided in that paper as to how those data were coded.

7 Dastur (2001) also found no difference between pregnant and non-pregnant women in UPSIT performance. Performance by odor was not reported in that study.

8 These data are in accord with a large retrospective self-report study by Cantoni et al. (1999) that was published only in abstract form. Approximately three-quarters of women reported that there were odors that smelled less pleasant during pregnancy (e.g., cigarettes, coffee, meat, food in general, diesel exhaust, and sweat). Less than a quarter of pregnant women reported that there were odors that smelled more pleasant (fruits, flowers, woodlands, and perfume).

9 USA Today, 12/6/2011

10 http://www.obesity.org/images/TOSpositionPaperlHCGrxObesityRevised12-12_-_Final_Approved_1-23-13.pdf.
